# Association between Hospital-Acquired Pneumonia and In-Hospital Mortality in Solid Organ Transplant Admissions: An Observational Analysis in Spain, 2004–2021

**DOI:** 10.3390/jcm12175532

**Published:** 2023-08-25

**Authors:** José M. de-Miguel-Yanes, Ana Lopez-de-Andres, Rodrigo Jimenez-Garcia, José Javier Zamorano-Leon, David Carabantes-Alarcon, Ricardo Omaña-Palanco, Valentín Hernández-Barrera, Jose Luis del-Barrio, Javier de-Miguel-Diez, Natividad Cuadrado-Corrales

**Affiliations:** 1 Internal Medicine Department, Hospital General Universitario Gregorio Marañón, Instituto de Investigación Sanitaria Gregorio Marañón (IiSGM), Universidad Complutense de Madrid, 28040 Madrid, Spain; josemaria.demiguel@salud.madrid.org; 2 Department of Public Health & Maternal and Child Health, Faculty of Medicine, Universidad Complutense de Madrid, 28040 Madrid, Spain; rodrijim@ucm.es (R.J.-G.); josejzam@ucm.es (J.J.Z.-L.); dcaraban@ucm.es (D.C.-A.); romana@ucm.es (R.O.-P.); mariancu@ucm.es (N.C.-C.); 3 Preventive Medicine and Public Health Teaching and Research Unit, Health Sciences Faculty, Rey Juan Carlos University, 28933 Alcorcón, Spain; valentin.hernandez@urjc.es (V.H.-B.); jose.delbarrio@urjc.es (J.L.d.-B.); 4 Respiratory Care Department, Hospital General Universitario Gregorio Marañón, Instituto de Investigación Sanitaria Gregorio Marañón (IiSGM), Universidad Complutense de Madrid, 28040 Madrid, Spain; javier.miguel@salud.madrid.org

**Keywords:** kidney transplant, liver transplant, heart transplant, lung transplant, hospital-acquired pneumonia, in-hospital mortality

## Abstract

(1) Background: To analyze the association between hospital-acquired pneumonia (HAP) and in-hospital mortality (IHM) during hospital admission for solid organ transplant in Spain during 2004–2021. (2) Methods: We used national hospital discharge data to select all hospital admissions for kidney, liver, heart, and lung transplants. We stratified the data according to HAP status. To examine time trends, we grouped data into three consecutive 6-year periods (2004–2009; 2010–2015; and 2016–2021). We assessed in-hospital mortality (IHM) in logistic regression analyses and obtained odds ratios (ORs) with their 95% confidence intervals (CIs). (3) Results: We identified a total of 71,827 transplants (45,262, kidney; 18,127, liver; 4734, heart; and 4598, lung). Two thirds of the patients were men. Overall, the rate of HAP during admission was 2.6% and decreased from 3.0% during 2004–2009 to 2.4% during 2016–2021. The highest rate of HAP corresponded to lung transplant (9.4%), whereas we found the lowest rate for kidney transplant (1.1%). Rates of HAP for liver and heart transplants were 3.8% and 6.3%, respectively. IHM was significantly lower during 2016–2021 compared to 2004–2009 for all types of transplants (ORs (CIs) = 0.65 (0.53–0.79) for kidney; 0.73 (0.63–0.84) for liver; 0.72 (0.59–0.87) for heart; and 0.39 (0.31–0.47) for lung). HAP was associated with IHM for all types of transplants (ORs (CIs) = 4.47 (2.85–9.08) for kidney; 2.96 (2.34–3.75) for liver; 1.86 (1.34–2.57) for heart; and 2.97 (2.24–3.94) for lung). (4) Conclusions: Rates of HAP during admission for solid organ transplant differ depending on the type of transplant. Although IHM during admission for solid organ transplant has decreased over time in our country, HAP persists and is associated with a higher IHM after accounting for potential confounding variables.

## 1. Introduction

Spain is a leading country in organ donation and transplant [1]. Our research group has previously evaluated the influence of clinical factors such as diabetes on outcomes during admission for solid organ transplants [2]. A deeper knowledge of factors associated with in-hospital mortality (IHM) shortly after transplant could inform clinical decisions and stimulate the implementation of strategies to improve the short-term prognosis of people undergoing solid organ transplant.

Our group has also dedicated research efforts to better understand the impact of hospital-acquired pneumonia (HAP) on IHM in other settings [3,4]. In the field of solid organ transplant, many papers have reported incidence rates of HAP and outcomes of patients diagnosed with HAP, but figures for a particular organ are quite variable in the literature and several of these studies are supported by unicentric databases [5]. Striking differences in incidence rates of HAP have been found to be related to the organ transplanted; lung transplant has been claimed to be associated with the highest rates of HAP [6]. 

Moreover, rates of HAP after transplant may have changed over time because of the evolution of the immunosuppression regimes followed by these patients [7] or perhaps due to a more active search of infection in these vulnerable people [8]. It is hard to find research papers that provide a bigger picture of the evolution of rates of HAP in people undergoing any type of solid organ transplant and its role on IHM after the procedure. Even in reviews on the topic, references including data from more than one institution that focus on time trends in incidence rates for more than one type of transplant are rare [9].

Therefore, we thought that if we could use data from a large, multicentric database covering a longer period than older research work, we might bring new information on the evolution of rates of HAP in patients undergoing solid organ transplants and provide updated evidence on the impact of the development of HAP on IHM during hospital admission for organ transplant. Thus, the evaluation of incidence rates of HAP, its impact on short-term survival, the analysis of IHM for each type of solid organ transplant and all of them combined, and the description of time trends in IHM during admission for transplant using national data spanning a long enough period was the main motivation to conduct this research. 

For this study, we used the Spanish National Hospital Discharge Data (SNHDD) [10] for the time period 2004–2021 to examine time trends in the number of transplants, rates of HAP, and outcomes during admission for kidney, liver, heart, and lung transplant among patients in Spain. We aimed to describe IHM rates to evaluate to what extent the development of HAP could influence the clinical course of patients receiving a solid organ transplant during the period of hospital admission for the performance of the procedure.

## 2. Materials and Methods

We performed a retrospective, observational study using the SNHDD, which compiles all public hospital data covering more than 95% of hospital admissions. Most solid organ transplants in Spain are carried out in public hospitals. For coding purposes, the SNHDD used the International Classification of Diseases, Ninth Revision, Clinical Modification (ICD-9-CM) until its transition to the International Classification of Diseases, Tenth Revision, Clinical Modification (ICD-10-CM) in 2015. Beyond clinical variables, it gathers information on up to 14 discharge diagnoses and up to 20 procedures performed during the index hospitalization. We collected data for 18 complete years (2004–2021).

### 2.1. Inclusion Criteria

We selected admissions for patients aged ≥18 years whose medical procedures included kidney transplant (ICD-9-CM codes 55.6, 55.61, and 55.69; ICD-10-CM code 0TYxxxx), liver transplant (ICD-9-CM codes 50.5, 50.51, and 50.59; ICD-10-CM code 0FY0), heart transplant (ICD-9-CM code 37.51; ICD-10-CM code 02YA0Zx), and lung transplant (ICD-9-CM codes 33.5, 33.50, 33.51, and 33.52; ICD-10-CM codes 0BYC, 0BYD, 0BYF, 0BYG, 0BYH, 0BYJ, 0BYK, 0BYL, and 0BYM) in any procedural field (Appendix A). Combined transplants contributed separate data for each category.

### 2.2. Study Variables

We identified all episodes of bacterial HAP, both associated and not associated with mechanical ventilation using ICD-9-CM codes 481–486 and 997.31 and ICD-10-CM codes J13-J18 and J95.851. 

We assessed the comorbidity of the population with the Charlson’s comorbidity index (CCI) [11]. The CCI applies to different disease categories, the scores of which are added to obtain an overall score for each patient. We grouped patients into three categories: low CCI (patients with no previously recorded disease), medium CCI (patients with one disease category), and high CCI (patients with two or more disease categories). 

We additionally retrieved data about microbiological isolations, in-hospital infections (e.g., cytomegalovirus infection), complications of the transplanted organs, leucopenia, urinary tract infection, cirrhosis (for liver transplant), hypogammaglobulinemia (for heart transplant) [12], and COVID-19 for the period 2016–2021 (ICD-10-CM code U07.1). The codes for these diagnoses can be found in Appendix A and Appendix B.

As main variables, we estimated the median of length of hospital stay (LOHS) and the IHM, defined by the proportion of patients who died during admission for each time period evaluated.

### 2.3. Statistical Analyses

We considered three time periods that included six consecutive years each (2004–2009, 2010–2015, and 2016–2021). We grouped patients into four age groups: ≤44, 45–54, 55–64, and ≥65 years old. We performed descriptive statistical analyses for all continuous variables and categories by stratifying admissions for kidney, liver, heart, and lung transplants according to HAP. Data are expressed as proportions and as means plus standard deviations or medians plus interquartile ranges for continuous variables. We compared continuous variables using the *t* test or the Mann–Whitney test and categorical variables using the chi-square test.

We performed logistic regression analyses on the entire study population to assess the influence of HAP taken as a covariate on IHM. The results are shown as odds ratios (ORs) with their 95% confidence intervals (CIs). For the multivariable analysis, we included those variables with a significant association with IHM in bivariate tests plus other study variables that, despite not being significant, were found to be important by other authors in previously published references in the field. Then, to decide which independent variables should remain in the final model, we used the Wald statistic after including them one by one. We compared consecutive models, which were created as new variables were incorporated, with the previous ones using the likelihood ratio test. Once we reached the final model, we checked for the eventual presence of linearity and interactions between the variables included in the model. We limited interaction tests to first-order (two by two) interactions. 

We used Stata version 10.1 (Stata, College Station, TX, USA) for the statistical analyses. We set statistical significance at *p* < 0.05 (2-tailed).

### 2.4. Ethical Aspects

Data confidentiality was maintained at all times. Patient identifiers were deleted before the database was provided to the authors. It is not possible to identify patients on an individual level, either in this article or in the database. Given the anonymous and mandatory nature of the dataset, it was not deemed necessary to obtain informed consent or an approval by an ethics committee, in accordance with the Spanish legislation.

## 3. Results

### 3.1. Hospital-Acquired Pneumonia during Admission in All Transplants Combined

A total of 71,827 transplants (67.0% among men) were performed in Spain during the 18-year period 2004–2021 in an increasingly older population (*p* < 0.001) (Table 1). 

**Figure 1:**
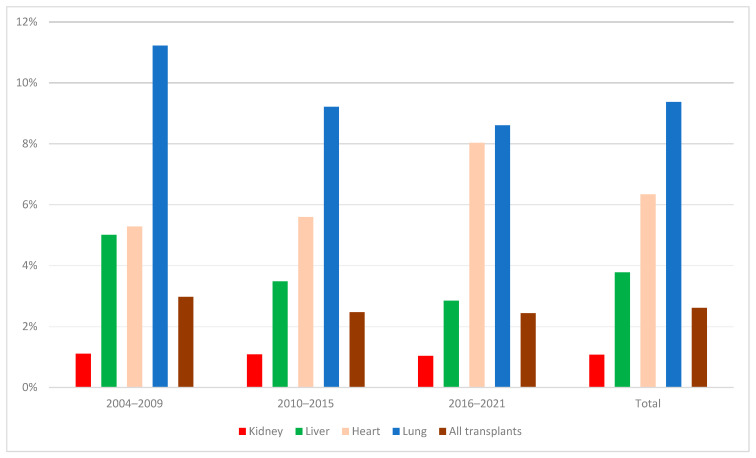
Rates of hospital-acquired pneumonia during admission for solid organ transplant in Spain distributed in 6-year periods (2004–2009, 2010–2015, and 2016–2021) and the total.

The overall rate of HAP during admission for solid organ transplants decreased from 3.0% during 2004–2009 to 2.4% during 2016–2021 (Figure 1). The highest rates of HAP corresponded to lung transplant (11.2% in 2004–2009 vs. 8.6% in 2016–2021). The lowest rates of HAP were for kidney transplant (around 1% during the whole period). We found decreasing rates of HAP for liver transplant (from 5.0% to 2.8%) but increasing rates for heart transplant (from 5.3% to 8.0%) when comparing the periods 2004–2009 vs. 2016–2021.

### 3.2. Hospital-Acquired Pneumonia during Admission for Kidney Transplant

Along the 18-year period, a total of 45,262 kidney transplants (64.0% among men) were carried out in Spain (Table 2). Age and comorbidity progressively increased in these patients. The risk of developing HAP was higher in the group aged ≥65 (*p* < 0.001) and with high CCI (*p* < 0.001), but no differences were found for sex (*p* = 0.787). People coded for HAP more often had complications of kidney transplant (*p* < 0.001), urinary tract infection (*p* = 0.005), cytomegalovirus infection (*p* < 0.001), and COVID-19 (*p* < 0.001). LOHS was significantly longer in people who developed HAP (27 ± 29 vs. 13 ± 10 days; *p* < 0.001). IHM did not change over time (*p* = 0.489) and was higher in people with a HAP diagnosis (14.2% vs. 1.2%; *p* < 0.001).

### 3.3. Hospital-Acquired Pneumonia during Admission for Liver Transplant

Between 2004 and 2021, a total of 18,127 liver transplants (73.2% among men) were performed in Spain (Table 3). Overall, age and comorbidity increased in this population. We found no differences between people who developed HAP vs. people without HAP regarding sex (*p* = 0.053), age (*p* = 0.673), or leucopenia (*p* = 0.748). People diagnosed with HAP more often had complications of liver transplant, urinary tract infection, and cytomegalovirus infection (all *p* values < 0.001), yet less often had cirrhosis (79.5% vs. 82%; *p* = 0.01). LOHS was significantly longer in people who developed HAP (35 ± 40 vs. 19 ± 17 days; *p* < 0.001). IHM was significantly higher among people who suffered HAP (20.3% vs. 6.8%; *p* < 0.001) but progressively lowered over time (from 27.0% to 13.1% in the periods 2004–2009 and 2016–2021, respectively; *p* < 0.001).

### 3.4. Hospital-Acquired Pneumonia during Admission for Heart Transplant

During the 18-year period evaluated, a total of 4734 heart transplants (75.1% among men) were performed in Spain (Table 4). People who were coded for HAP more often had urinary tract infection (*p* = 0.044) and cytomegalovirus infection (<0.001), but we found no differences for the remaining variables studied, including hypogammaglobulinemia (*p* = 0.989). LOHS was significantly longer in people who developed HAP (60 ± 59 vs. 29 ± 34 days; *p* < 0.001). IHM did not change over time (*p* = 0.236) and was higher in people diagnosed with HAP (28.7% vs. 16.1%; *p* < 0.001).

### 3.5. Hospital-Acquired Pneumonia during Admission for Lung Transplant

Between 2004 and 2021, a total of 4598 lung transplants (63.6% among men) were performed in Spain (Table 5). People who developed HAP more often had complications of lung transplant (*p* < 0.001), urinary tract infection (*p* = 0.002), and cytomegalovirus infection (*p* = 0.032), but we found no differences between the two groups for the remaining variables studied. LOHS was significantly longer in people who developed HAP (56 ± 56 vs. 34 ± 27 days; *p* < 0.001). IHM decreased over time (from 50.4% in the period 2004–2009 to 19.7% in the period 2016–2021; *p* < 0.001) and was higher among people who were diagnosed with HAP (29.7% vs. 13.3%; *p* < 0.001).

### 3.6. Factors Associated with In-Hospital Mortality during Admission for Solid Organ Transplant in Spain, 2004–2021

We could see no significant associations for any of the linearity or interaction analyses. When HAP was included in the IHM analysis as a covariate, we found that advanced age was a risk factor for IHM in all but lung transplants (Table 6). Female sex was associated with a higher IHM in people who underwent kidney (OR (CI) = 1.23 (1.04–1.46)) or heart transplant (OR (CI) = 1.24 (1.04–1.48)), whilst no significant effect of sex was seen for liver or lung transplant. 

Overall, the most frequently coded microorganism was *Pseudomonas aeruginosa*, especially in patients who underwent a lung transplant (15.3% of the isolations in this type of transplant; Table S1). However, a *Pseudomonas aeruginosa* code was associated with a higher IHM only among people undergoing a heart transplant (OR (CI) = 3.15 (1.33–7.49)).

IHM was significantly lower during the period 2016–2021 compared to 2004–2009 for all transplants analyzed (ORs (CIs) for kidney, liver, heart, and lung transplants were 0.65 (0.53–0.79), 0.73 (0.63–0.84), 0.72 (0.59–0.87), and 0.39 (0.31–0.47), respectively). 

HAP was a significant risk factor for IHM in all transplants evaluated (ORs (CIs) for kidney, liver, heart, and lung transplants were 4.47 (2.85–9.08), 2.96 (2.34–3.75), 1.86 (1.34–2.57), and 2.97 (2.24–3.94), respectively).

## 4. Discussion

Here, we found the highest rates of HAP among people undergoing lung transplant and the lowest rates among people undergoing kidney transplant. Lung transplant recipients are at greatest risk for the development of pneumonia due to factors more specific to this kind of transplant, such as impaired mucociliary function and cough mechanisms, gastroparesis, or need for prolonged mechanical ventilation [13]. Both the native and the donor lung can be colonized with pathogenic bacteria before the procedure [14], though other researchers have challenged this pathogenic mechanism [13]. At the other end, it has long been known that kidney transplant is the solid organ transplant associated with the lowest rates of pneumonia [15].

We do not have a clear explanation for the increasing rates of HAP among heart transplant subjects. Previous research has described that up to three-quarters of the pneumonias after heart transplant have their onset within the three-month period after the procedure [16]. Prolonged mechanical ventilation, blood transfusion [17], or use of extracorporeal membrane oxygenation [18] have all been associated with higher rates of HAP in patients undergoing heart surgery and could be some of the reasons to explain this finding. We do not believe that a higher incidence of urinary tract infection or a more frequent cytomegalovirus coding fully explains these results because these conditions were not associated with an increased incidence of HAP for the other types of transplants. Alternatively, a more meticulous microbiological search of infective complications after heart transplant in recent years [5] with the use of newer molecular techniques as seen for hematopoietic transplants [19] or even the more frequent use of computerized tomography [20] might have introduced a bias towards a more frequent HAP coding in this group despite uniformity in the diagnostic criteria. We had no access to variables such as the etiology of the end-stage heart failure or the degree of multiple organ damage before transplant (renal failure or pulmonary function) that might have contributed to the higher incidence of HAP seen in people aged under 45 years undergoing a heart transplant. Remarkably, another important variable that only operated during the period 2016–2021 was COVID-19, which could have posed a distinctive pneumonia risk only after heart transplant [21].

We identified age as a risk factor for HAP after a kidney transplant, in a similar fashion to what other authors have reported [22]. In our dataset, kidney transplant recipients who developed HAP were on average three years older than people who did not develop HAP. It is not clear why we detected this difference only after kidney transplant as age differences between people affected and not affected by HAP were not significant for the other types of transplants. Accordingly, other studies have also failed to detect age differences between people having HAP vs. no HAP after heart [23], lung [13], and liver transplants [24], but these studies included a much lower number of patients than ours and their negative results may have simply been due to a lack of statistical power.

In the survival analysis that we conducted, age was associated with a higher IHM in all groups except lung transplant. Certainly, compared with patients younger than 45 years, kidney transplant recipients older than 45 years, liver transplant recipients older than 64 years, and heart transplant recipients older than 54 years had lower in-hospital survival rates. Previous reports found comparable results in the short term after these mentioned types of transplants [25,26,27]. In our study, after accounting for potential confounders, we identified no significant differences in IHM between age groups during admission for lung transplant. In Spain, Rello et al. described that age independently predicted short-term mortality after lung transplant in a multicentric study that included data from 272 patients [28]. Nevertheless, other authors have underscored the influence of a complex constellation of factors rather than age per se, at least on the long-term survival after lung transplant [29]. Variables such as matching donor quality to recipient severity may have a greater impact than age on short-term survival after lung transplant [30].

In our database, a code for urinary tract infection was associated with higher rates of HAP after any type of transplant in the bivariate analyses. We can only speculate that previous use of antibiotics can select a more aggressive bacterial flora that triggers a more severe pulmonary infection. Instead, it could be that flora colonizing the urinary tract colonizes the respiratory mucosa too. The presence of cytomegalovirus infection is associated with a higher rate of HAP, as described by previous reports [5,31]. However, this association was not confirmed in the multivariable analysis.

A *Pseudomonas aeruginosa* code was more frequent among patients undergoing lung transplant but was associated with an increased IHM only after heart transplant. Pulmonologists may be more aware of the impact of colonization by *Pseudomonas aeruginosa* on graft survival and lung function and are more sensitive to eradicating it after lung transplant, eventually reducing its impact on mortality [32,33]. *Pseudomonas aeruginosa* infection has been claimed to worsen prognosis after heart surgery [34], but we have found no references to support a differential effect on IHM distinctively after heart transplant compared to other types of transplants.

IHM was significantly higher in people who developed HAP during admission for all types of solid organ transplants. The increased IHM associated with the onset of HAP ranged from almost 2-fold higher after heart transplant to more than 4-fold higher after kidney transplant. A high index of suspicion for pneumonia is necessary in a solid organ transplant recipient [35]. Diagnostic strategies must consider numerous factors, including post-transplant timing, degree of immunosuppression, environmental/community/hospital exposures, and seasonal epidemiology [36]. Failure to do so may worsen the prognosis. Unfortunately, we had no access to information about antibiotics use, which should be determined by local epidemiology and resistance patterns. We might speculate that HAP has a higher impact on IHM because it is less commonly found after kidney transplant, and the diagnosis can therefore be delayed for this transplant. However, a truly higher impact on IHM for a specific type of transplant cannot be dismissed. Causative agents may differ, and the impact of the infection on graft function or its complications may not be homogeneous [36].

Our findings also showed that IHM significantly decreased over time during admission for all types of solid organ transplants. Notably, evidence regarding time trends of IHM during admission for solid organ transplant is scarce. Indeed, most studies have focused on the long term. Awan et al. published results showing decreasing mortality rates over time during the 12 months after a kidney transplant in the United States, mainly driven by a reduction in cardiovascular mortality [37]. The reduction in mortality over time seen after a kidney transplant seems to be more remarkable in the short term than in the long term [38]. This conclusion has also been outlined after a liver transplant [39]. A better control of comorbidities may reduce short-term mortality after a liver transplant but may have a lesser impact on long-term mortality [40]. The scores used to predict short-term outcomes after heart transplant include recipients’ specific characteristics such as dialysis, extracorporeal membrane oxygenation, durable left-ventricle assist device support, intra-aortic balloon pump, use of inotropes at listing, or days on the waiting list [41]. Short-term reductions in IHM after heart transplant could involve recent changes in all these factors [42]. Lastly, modifications in donor selection practices, organ preservation, perioperative management, or better treatment of postoperative complications might explain the improvement in short-term mortality after lung transplant as well [43]. Regrettably, the design of our study does not allow us to establish the reasons for our findings.

The strength of our findings lies in the large sample size, with data from over 71,000 solid organ transplants and over 1800 episodes of HAP; the 18-year recruitment period covering the population of an entire country; and the standardized methodology, which has been used for research purposes earlier. Nevertheless, we should point out some limitations. First, we did not differentiate between ventilator-associated HAP and no-ventilator-associated HAP. Second, our data source was the SNHDD, an administrative database that relies on manual coding on behalf of administrative staff. We cannot rule out that coding practices may have changed over time. Moreover, inaccuracies in secondary diagnosis coding are possible, but we believe that mistakes in coding procedures such as organ transplants are not probable. Third, the limited number of COVID-19 codes during admission for transplants (total N = 83 cases) precludes drawing any conclusion about the impact of COVID-19 in this population. We must recall that the way to report confirmed SARS-Cov-2 infection only became uniform after 1 July 2020 [44]. Fourth, anonymity might limit the extraction of some pieces of information (e.g., people who moved from one hospital to another would appear twice, though this circumstance would be unusual after an admission for an organ transplant). Fifth, residual confounding that was not accounted for could have influenced the results of the multivariable analysis of the factors associated with in-hospital mortality.

## 5. Conclusions

In summary, we found decreasing IHM over time during admission for kidney, liver, heart, and lung transplants in Spain during the period 2004–2021. IHM was significantly higher in people who developed HAP during admission for solid organ transplant. Randomized clinical trials should be designed for both prevention and treatment of pneumonia after each type of transplant.

## Figures and Tables

**Table 1:** Sociodemographic and clinical characteristics of patients who underwent a solid organ transplant in Spain from 2004 to 2021 according to the presence of a diagnosis of hospital-acquired pneumonia (HAP).

		2004–2009		2010–2015		2016–2021		TOTAL		*p* Values	
		No HAP	HAP	No HAP	HAP	No HAP	HAP	No HAP	HAP	HAP vs. No HAP	HAP Trend
Number of transplants		20,881	640	22,464	568	26,608	666	69,953	1874		
Male sex, n (%)		13,807 (66.1)	444 (69.4)	15,137 (67.4)	387 (68.1)	17,867 (67.2)	466 (70.0)	46,811 (66.9)	1297 (69.2)	0.037	0.512
Female sex, n (%)		7074 (33.9)	196 (30.6)	7327 (32.6)	181 (31.9)	8741 (32.9)	200 (30.0)	23,142 (33.1)	577 (30.8)	0.037	0.512
Age, mean (SD)		49.7 (15.3)	49.7 (15.9)	52.0 (14.9)	53.0 (13.6)	54.1 (15.4)	54.2 (14.9)	52.1 (15.3)	52.3 (15.0)	0.560	<0.001
Age < 45 years, n (%)		6366 (30.5)	175 (27.3)	5586 (24.9)	109 (19.2)	5599 (21.0)	121 (18.2)	17,551 (25.1)	405 (21.6)	0.001	0.002
Age 45–54 years, n (%)		5298 (25.4)	161 (25.2)	5660 (25.2)	154 (27.1)	5527 (20.8)	123 (18.5)	16,485 (23.6)	438 (23.4)	0.846	0.007
Age 55–64 years, n (%)		6275 (30.1)	219 (34.2)	6962 (31.0)	215 (37.9)	8681 (32.6)	281 (42.2)	21,918 (31.3)	715 (38.2)	<0.001	0.404
Age ≥ 65 years, n (%)		2942 (14.1)	85 (13.3)	4256 (19.0)	90 (15.9)	6801 (25.6)	141 (21.2)	13,999 (20.0)	316 (16.9)	0.001	0.141
CCI, mean (SD)		1.5 (0.8)	1.5 (0.9)	1.5 (0.8)	1.4 (0.8)	1.6 (0.9)	1.7 (1.1)	1.6 (0.8)	1.5 (1.0)	0.173	<0.001
Low CCI, n (%)		714 (3.4)	51 (8.0)	707 (3.2)	61 (10.7)	1092 (4,1)	70 (10.5)	2513 (3.6)	182 (9.7)	<0.001	0.179
Medium CCI, n (%)		11,898 (57.0)	328 (51.2)	12,150 (54.0)	290 (51.1)	12,709 (47.8)	254 (38.1)	36,757 (52.6)	872 (46.5)	<0.001	0.003
High CCI, n (%)		8269 (39.6)	261 (40.8)	9607 (42.8)	217 (38.2)	12,807 (48.1)	342 (51.4)	30,683 (43.9)	820 (43.8)	0.928	0.012
Complications	No	14.869 (71.2)	347 (54.2)	16,123 (71.8)	312 (54.9)	19,115 (71.8)	355 (53.3)	50,107 (71.6)	1014 (54.1)	<0.001	<0.001
	Yes	6012 (28.8)	293 (45.8)	6341 (28.2)	256 (45.1)	7493 (28.2)	311 (46.7)	19,846 (28.4)	860 (45.9)		
Urinary tract infection	No	18,815 (90.1)	572 (89.4)	19,992 (89.0)	513 (90.3)	24,024 (90.3)	593 (89.0)	62,831 (89.8)	1678 (89.6)	0.695	0.251
	Yes	2066 (9.9)	68 (10.6)	2472 (11.0)	55 (9.7)	2584 (9.7)	73 (11.0)	7122 (10.2)	196 (10.5)		
Cytomegalovirus	No	20,541 (98.4)	595 (93.0)	22,030 (98.1)	543 (95.6)	25,832 (97.1)	601 (90.3)	68,403 (97.8)	1739 (92.8)	<0.001	0.024
	Yes	340 (1.6)	45 (7.0)	434 (1.9)	25 (4.4)	776 (2.9)	65 (9.8)	1550 (2.2)	135 (7.2)		
Leucopenia	No	20,830 (99.8)	635 (99.3)	22,217 (98.9)	566 (99.7)	26,408 (99.3)	661 (99.3)	69,455 (99.3)	1862 (99.4)	0.716	0.150
	Yes	51 (0.2)	5 (0.8)	247 (1.1)	2 (0.4)	200 (0.8)	5 (0.8)	498 (0.7)	12 (0.6)		
COVID-19	No					26,531 (99.7)	660 (99.1)	69,876 (99.9)	1868 (99.7)	0.021	
	Yes					77 (0.3)	6 (0.9)	77 (0.1)	6 (0.3)		
LOHS, median (IQR)		17 (16)	39 (45)	16 (15)	38 (46)	15 (15)	43 (48)	16 (15)	40 (47)	<0.001	<0.001
IHM, n (%)		1072 (5.1)	186 (29.1)	887 (4.0)	108 (19.0)	956 (3.6)	119 (17.9)	2915 (4.2)	413 (22.0)	<0.001	<0.001

HAP: hospital-acquired pneumonia. SD: standard deviation. CCI: Charlson comorbidity index. CCI applies to different disease categories, the scores of which are added to obtain an overall score for each patient. We have grouped patients into three categories: low CCI (patients with no previously recorded disease), medium CCI (patients with one category), and high CCI (patients with two or more disease categories). LOHS: length of hospital stay. IQR: interquartile range. IHM: in-hospital mortality.

**Table 2:** Sociodemographic and clinical characteristics of patients who underwent a kidney transplant in Spain from 2004 to 2021 according to the presence of a diagnosis of hospital-acquired pneumonia (HAP).

		2004–2009		2010–2015		2016–2021		TOTAL		*p* Values	
		No HAP	HAP	No HAP	HAP	No HAP	HAP	No HAP	HAP	HAP vs. No HAP	HAP Trend
Number of transplantations		12,836	144	14,291	157	17,649	185	44,776	486		
Male sex, n (%)		8044 (62.7)	92 (63.4)	9136 (64.0)	98 (62.4)	11,485 (65.1)	124 (67.0)	28,665 (64.0)	314 (64.6)	0.787	0.659
Female sex, n (%)		4792 (37.3)	52 (36.1)	5155 (36.07)	59 (37.6)	6164 (34.9)	61 (33.0)	16,111 (36.0)	172 (35.4)	0.787	0.659
Age, mean (SD)		49.5 (15.2)	52.9 (15.0)	52.1 (15.0)	56.7 (13.6)	54.7 (15.3)	56.8 (15.2)	52.4 (15.3)	55.6 (14.7)	<0.001	0.030
Age < 45 years, n (%)		4442 (34.6)	43 (29.9)	4046 (28.3)	28 (17.8)	4001 (22.7)	29 (15.7)	12,489 (27.9)	100 (20.6)	<0.001	0.004
Age 45–54 years, n (%)		3048 (23.8)	24 (16.7)	3391 (23.7)	33 (21.0)	3722 (21.1)	31 (16.8)	10,161 (22.7)	88 (18.1)	0.016	0.515
Age 55–64 years, n (%)		3225 (25.1)	34 (23.6)	3629 (25.4)	40 (25.5)	4697 (26.6)	65 (35.1)	11,551 (25.8)	139 (28.6)	0.160	0.041
Age ≥ 65 years, n (%)		2121 (16.5)	43 (29.9)	3225 (22.6)	56 (35.7)	5229 (29.6)	60 (32.4)	10,575 (23.6)	159 (32.7)	<0.001	0.559
CCI, mean (SD)		1.4 (0.7)	1.6 (0.8)	1.5 (0.7)	1.6 (0.7)	1.6 (0.8)	1.9 (1.0)	1.5 (0.7)	1.7 (0.9)	<0.001	0.001
Low CCI, n (%)		126 (1.0)	4 (2.8)	76 (0.5)	4 (2.6)	180 (1.0)	2 (1.1)	382 (0.9)	10 (2.1)	0.004	0.488
Medium CCI, n (%)		8386 (65.3)	70 (48.6)	8727 (61.1)	77 (49.0)	9445 (53.5)	78 (42.2)	26,558 (59.3)	225 (46.3)	<0.001	0.357
High CCI, n (%)		4324 (33.7)	70 (48.6)	5488 (38.4)	76 (48.4)	8024 (45.5)	105 (56.8)	17,836 (39.8)	251 (51.7)	<0.001	0.210
Complications	No	9321 (72.6)	76 (52.8)	10,524 (73.6)	87 (55.4)	13,073 (74.1)	99 (53.5)	32,918 (73.5)	262 (53.9)	<0.001	0.892
	Yes	3515 (27.4)	68 (47.2)	3767 (26.4)	70 (44.6)	4576 (25.9)	86 (46.5)	11,858 (26.5)	224 (46.1)		
Urinary tract infection	No	11,063 (86.2)	116 (80.6)	12,207 (85.4)	127 (80.9)	15,462 (87.6)	156 (84.3)	38,732 (86.5)	399 (82.1)	0.005	0.603
	Yes	1773 (13.8)	28 (19.4)	2084 (14.6)	30 (19.1)	2187 (12.4)	29 (15.7)	6044 (13.5)	87 (17.9)		
Cytomegalovirus	No	12,708 (99.0)	136 (94.4)	14,123 (98.8)	151 (96.2)	17,281 (97.9)	174 (94.1)	44,112 (98.5)	461 (94.9)	<0.001	0.652
	Yes	128 (1.0)	8 (5.6)	168 (1.2)	6 (3.8)	368 (2.1)	11 (6.0)	664 (1.5)	25 (5.1)		
Leucopenia	No	12,800 (99.7)	143 (99.3)	14,131 (98.9)	156 (99.4)	17,505 (99.2)	185 (100)	44,436 (99.2)	484 (99.6)	0.378	0.538
	Yes	36 (0.3)	1 (0.7)	160 (1.1)	1 (0.6)	144 (0.8)	0 (0)	340 (0.8)	2 (0.4)		
COVID-19	No					17,604 (99.7)	181 (97.8)	44,731 (99.9)	482 (99.2)	<0.001	
	Yes					45 (0.3)	4 (2.2)	45 (0.1)	4 (0.8)		
LOHS, median (IQR)		14 (12)	30 (34)	13 (10)	25 (29)	13 (10)	27 (25)	13 (10)	27 (29)	<0.001	0.284
IHM, n (%)		176 (1.4)	22 (15.3)	146 (1.0)	18 (11.5)	211 (1.2)	29 (15.7)	533 (1.2)	69 (14.2)	<0.001	0.489

HAP: hospital-acquired pneumonia. SD: standard deviation. CCI: Charlson comorbidity index. CCI applies to different disease categories, the scores of which are added to obtain an overall score for each patient. We have grouped patients into three categories: low CCI (patients with no previously recorded disease), medium CCI (patients with one category), and high CCI (patients with two or more disease categories). LOHS: length of hospital stay. IQR: interquartile range. IHM: in-hospital mortality.

**Table 3:** Sociodemographic and clinical characteristics of patients who underwent a liver transplant in Spain from 2004 to 2021 according to the presence of a diagnosis of hospital-acquired pneumonia (HAP).

		2004–2009		2010–2015		2016–2021		TOTAL		*p* Values	
		No HAP	HAP	No HAP	HAP	No HAP	HAP	No HAP	HAP	HAP vs. No HAP	HAP Trend
Number of transplantations		5767	304	5661	204	6015	176	17,443	684		
Male sex, n (%)		4128 (71.6)	207 (68.1)	4255 (75.2)	141 (69.1)	4414 (73.4)	131 (74.4)	12,797 (73.4)	479 (70.0)	0.053	0.325
Female sex, n (%)		1639 (28.4)	97 (31.9)	1406 (24.8)	63 (30.9)	1601 (26.6)	45 (25.6)	4646 (26.6)	205 (30.0)	0.053	0.325
Age, mean (SD)		50.3 (15.1)	49.4 (16.2)	52.0 (14.6)	52.9 (12.4)	53.6 (15.5)	56.3 (12.1)	52.0 (15.2)	52.2 (14.4)	0.673	<0.001
Age < 45 years, n (%)		1308 (22.7)	76 (25)	961 (17.0)	29 (14.2)	911 (15.2)	17 (9.7)	3180 (18.2)	122 (17.8)	0.793	<0.001
Age 45–54 years, n (%)		1709 (29.6)	84 (27.6)	1723 (30.4)	67 (32.8)	1272 (21.2)	34 (19.3)	4704 (27.0)	185 (27.1)	0.964	0.012
Age 55–64 years, n (%)		2154 (37.4)	111 (36.5)	2238 (39.5)	87 (42.7)	2682 (44.6)	84 (47.7)	7074 (40.6)	282 (41.2)	0.725	0.049
Age ≥ 65 years, n (%)		596 (10.3)	33 (10.9)	739 (13.1)	21 (10.3)	1150 (19.1)	41 (23.3)	2485 (14.3)	95 (13.9)	0.793	<0.001
CCI, mean (SD)		1.7 (0.9)	1.6 (0.8)	1.8 (0.9)	1.6 (0.9)	1.8 (1.0)	2.0 (1.0)	1.8 (0.9)	1.7 (0.9)	0.008	<0.001
Low CCI, n (%)		239 (4.1)	12 (4.0)	214 (3.8)	10 (4.9)	355 (5.9)	5 (2.8)	808 (4.6)	27 (4.0)	0.402	0.589
Medium CCI, n (%)		2389 (41.4)	160 (52.6)	2208 (39.0)	106 (52.0)	1988 (33.1)	57 (32.4)	6585 (37.8)	323 (47.2)	<0.001	<0.001
High CCI, n (%)		3139 (54.4)	132 (43.4)	3239 (57.2)	88 (43.1)	3672 (61.1)	114 (64.8)	10,050 (57.6)	334 (48.8)	<0.001	<0.001
Complications	No	4151 (72.0)	187 (61.5)	4138 (73.1)	140 (68.6)	4475 (74.4)	118 (67.1)	12,764 (73.2)	445 (65.1)	<0.001	0.209
	Yes	1616 (28.0)	117 (38.5)	1523 (26.9)	64 (31.4)	1540 (25.6)	58 (33.0)	4679 (26.8)	239 (34.9)		
Urinary tract infection	No	5540 (96.1)	276 (90.8)	5369 (94.8)	187 (91.7)	5747 (95.5)	158 (89.8)	16,656 (95.5)	621 (90.8)	<0.001	0.817
	Yes	227 (3.9)	28 (9.2)	292 (5.2)	17 (8.3)	268 (4.5)	18 (10.2)	787 (4.5)	63 (9.2)		
Cytomegalovirus	No	5613 (97.3)	278 (91.5)	5467 (96.6)	191 (93.6)	5748 (95.6)	152 (86.4)	16,828 (96.5)	621 (90.8)	<0.001	0.044
	Yes	154 (2.7)	26 (8.6)	194 (3.4)	13 (6.4)	267 (4.4)	24 (13.6)	615 (3.5)	63 (9.2)		
Leucopenia	No	5756 (99.8)	303 (99.7)	5602 (99.0)	204 (100)	5965 (99.2)	173 (98.3)	17,323 (99.3)	680 (99.4)	0.748	0.069
	Yes	11 (0.2)	1 (0.3)	59 (1.0)	0 (0)	50 (0.8)	3 (1.7)	120 (0.7)	4 (0.6)		
Cirrhosis	No	1069 (18.5)	71 (23.4)	963 (17.0)	47 (23.0)	1107 (18.4)	22 (12.5)	3139 (18.0)	140 (20.5)	0.010	0.010
	Yes	4698 (81.5)	233 (76.6)	4698 (83.0)	157 (77.0)	4908 (81.6)	154 (87.5)	14,304 (82.0)	544 (79.5)		
COVID-19	No					5991 (99.6)	175 (99.4)	17,419 (99.9)	683 (99.9)	0.953	
	Yes					24 (0.4)	1 (0.6)	24 (0.1)	1 (0.1)		
LOHS, median (IQR)		21 (19)	39 (45)	20 (18)	33 (38)	18 (17)	34 (35)	19 (17)	35 (40)	<0.001	0.039
IHM, n (%)		451 (7.8)	82 (27.0)	370 (6.5)	34 (16.7)	366 (6.1)	23 (13.1)	1187 (6.8)	139 (20.3)	<0.001	<0.001

HAP: hospital-acquired pneumonia. SD: standard deviation. CCI: Charlson comorbidity index. CCI applies to different disease categories, the scores of which are added to obtain an overall score for each patient. We have grouped patients into three categories: low CCI (patients with no previously recorded disease), medium CCI (patients with one category), and high CCI (patients with two or more disease categories). LOHS: length of hospital stay. IQR: interquartile range. IHM: in-hospital mortality.

**Table 4:** Sociodemographic and clinical characteristics of patients who underwent a heart transplant in Spain from 2004 to 2021 according to the presence of a diagnosis of hospital-acquired pneumonia (HAP).

		2004–2009		2010–2015		2016–2021		TOTAL		*p* Values	
		No HAP	HAP	No HAP	HAP	No HAP	HAP	No HAP	HAP	HAP vs. No HAP	HAP Trend
Number of transplantations		1561	87	1350	80	1523	133	4434	300		
Male sex, n (%)		1209 (77.5)	69 (79.3)	1024 (75.9)	62 (77.5)	1092 (71.7)	100 (75.2)	3325 (75.0)	231 (77.0)	0.436	0.771
Female sex, n (%)		352 (22.6)	18 (20.7)	326 (24.2)	18 (22.5)	431 (28.3)	33 (24.8)	1109 (25.0)	69 (23.0)	0.436	0.771
Age, mean (SD)		50.5 (16.4)	47.6 (17.6)	50.6 (16.1)	50.4 (15.9)	48.6 (17.9)	48.7 (17.8)	49.9 (16.9)	48.8 (17.3)	0.297	0.581
Age < 45 years, n (%)		381 (24.4)	25 (28.7)	337 (25.0)	20 (25.0)	437 (28.7)	42 (31.6)	1155 (26.1)	87 (29.0)	0.261	0.590
Age 45–54 years, n (%)		378 (24.22)	22 (25.3)	314 (23.3)	21 (26.3)	328 (21.5)	26 (19.6)	1020 (23.0)	69 (23.0)	0.999	0.443
Age 55–64 years, n (%)		601 (38.5)	32 (36.8)	494 (36.6)	31 (38.8)	536 (35.2)	48 (36.1)	1631 (36.8)	111 (37.0)	0.940	0.926
Age ≥ 65 years, n (%)		201 (12.9)	8 (9.2)	205 (15.2)	8 (10.0)	222 (14.6)	17 (12.3)	628 (14.2)	33 (11.0)	0.126	0.669
CCI, mean (SD)		1.6 (0.9)	1.7 (0.9)	1.7 (1.0)	1.5 (0.8)	2.1 (1.1)	2.0 (1.1)	1.8 (1.0)	1.8 (1.0)	0.545	0.005
Low CCI, n (%)		109 (7.0)	5 (5.8)	82 (6.1)	5 (6.3)	73 (4.8)	7 (5.3)	264 (6.0)	17 (5.7)	0.838	0.955
Medium CCI, n (%)		655 (42.0)	35 (40.2)	541 (40.1)	36 (45.0)	394 (25.9)	41 (30.8)	1590 (35.9)	112 (37.3)	0.607	0.094
High CCI, n (%)		797 (51.1)	47 (54.0)	727 (53.9)	39 (48.8)	1056 (69.3)	85 (63.9)	2580 (58.2)	171 (57.0)	0.687	0.077
Complications	No	1117 (71.6)	55 (63.2)	887 (65.7)	52 (65.0)	1006 (66.1)	85 (63.9)	3010 (67.9)	192 (64.0)	0.164	0.971
	Yes	444 (28.4)	32 (36.8)	463 (34.3)	28 (35.0)	517 (33.9)	48 (36.1)	1424 (32.1)	108 (36.0)		
Urinary tract infection	No	1498 (96.0)	80 (91.9)	1272 (94.2)	76 (95.0)	1394 (91.5)	117 (88.0)	4164 (93.9)	273 (91.0)	0.044	0.207
	Yes	63 (4.0)	7 (8.1)	78 (5.8)	4 (5.0)	129 (8.5)	16 (12.0)	270 (6.1)	27 (9.0)		
Cytomegalovirus	No	1524 (97.6)	83 (95.4)	1302 (96.4)	78 (97.5)	1435 (94.2)	114 (85.7)	4261 (96.1)	275 (91.7)	<0.001	0.003
	Yes	37 (2.4)	4 (4.6)	48 (3.6)	2 (2.5)	88 (5.8)	19 (14.3)	173 (3.9)	25 (8.3)		
Leucopenia	No	1561 (100)	86 (98.8)	1341 (99.3)	80 (100)	1514 (99.4)	132 (99.2)	4416 (99.6)	298 (99.3)	0.500	0.651
	Yes	0 (0)	1 (1.2)	9 (0.7)	0 (0)	9 (0.6)	1 (0.8)	18 (0.4)	2 (0.7)		
Hypogammaglobulinemia	No	1555 (99.6)	86 (98.8)	1347 (99.8)	80 (100)	1517 (99.6)	133 (100)	4419 (99.7)	299 (99.7)	0.989	0.293
	Yes	6 (0.4)	1 (1.2)	3 (0.2)	0 (0)	6 (0.4)	0 (0)	15 (0.3)	1 (0.3)		
COVID-19	No					1518 (99.7)	133 (100)	4429 (99.9)	300 (100)	0.561	
	Yes					5 (0.3)	0 (0)	5 (0.1)	0 (0)		
LOHS, median (IQR)		26 (29)	48 (56)	28 (34)	57 (56)	33 (43)	73 (55)	29 (34)	60 (59)	<0.001	<0.001
IHM, n (%)		275 (17.6)	30 (34.5)	226 (16.7)	24 (30.0)	211 (13.9)	32 (24.1)	712 (16.1)	86 (28.7)	<0.001	0.236

HAP: hospital-acquired pneumonia. SD: standard deviation. CCI: Charlson comorbidity index. CCI applies to different disease categories, the scores of which are added to obtain an overall score for each patient. We have grouped patients into three categories: low CCI (patients with no previously recorded disease), medium CCI (patients with one category), and high CCI (patients with two or more disease categories). LOHS: length of hospital stay. IQR: interquartile range. IHM: in-hospital mortality.

**Table 5:** Sociodemographic and clinical characteristics of patients who underwent a lung transplant in Spain from 2004 to 2021 according to the presence of a diagnosis of hospital-acquired pneumonia (HAP).

		2004–2009		2010–2015		2016–2021		TOTAL		*p* Values	
		No HAP	HAP	No HAP	HAP	No HAP	HAP	No HAP	HAP	HAP vs. No HAP	HAP Trend
Number of transplantations		894	113	1330	135	1943	183	4167	431		
Male sex, n (%)		554 (62.0)	81 (71.7)	834 (62.7)	92 (68.1)	1246 (64.1)	119 (65.0)	2634 (63.2)	292 (67.8)	0.062	0.489
Female sex, n (%)		340 (38.0)	32 (28.3)	496 (37.3)	43 (31.9)	697 (35.9)	64 (35.0)	1533 (36.8)	139 (32.3)	0.062	0.489
Age, mean (SD)		47.3 (15.2)	48.5 (14.5)	51.9 (13.4)	50.6 (12.9)	54.0 (12.9)	53.5 (13.2)	51.9 (13.8)	51.3 (13.6)	0.382	0.007
Age < 45 years, n (%)		284 (31.8)	33 (2.2)	285 (21.4)	32 (23.7)	352 (18.1)	35 (19.1)	921 (22.1)	100 (23.2)	0.601	0.135
Age 45–54 years, n (%)		219 (24.5)	31 (27.4)	283 (21.3)	38 (28.2)	333 (17.1)	34 (18.6)	835 (20.0)	103 (23.9)	0.058	0.084
Age 55–64 years, n (%)		356 (39.8)	44 (38.9)	662 (49.8)	58 (43.0)	975 (50.2)	91 (49.7)	1993 (47.8)	193 (44.8)	0.228	0.169
Age ≥ 65 years, n (%)		35 (3.9)	5 (4.42)	100 (7.5)	7 (5.2)	283 (14.6)	23 (12.6)	418 (10.0)	35 (8.1)	0.205	0.014
CCI, mean (SD)		0.9 (0.7)	0.9 (0.7)	1.0 (0.8)	0.8 (0.7)	1.0 (0.8)	1.0 (0.9)	1.0 (0.8)	0.9 (0.8)	0.150	0.170
Low CCI, n (%)		243 (27.2)	31 (27.4)	341 (25.7)	44 (32.6)	515 (26.5)	57 (31.2)	1099 (26.4)	132 (30.6)	0.058	0.667
Medium CCI, n (%)		504 (56.4)	64 (56.6)	707 (53.2)	72 (53.3)	1016 (52.3)	83 (45.4)	2227 (53.4)	219 (50.8)	0.297	0.131
High CCI, n (%)		147 (16.4)	18 (16.0)	282 (21.2)	19 (14.1)	412 (21.2)	43 (23.5)	841 (20.2)	80 (18.6)	0.423	0.072
Complications	No	492 (55.0)	39 (34.5)	762 (57.3)	41 (30.4)	1106 (56.9)	71 (38.8)	2360 (56.6)	151 (35.0)	<0.001	0.295
	Yes	402 (45.0)	74 (65.5)	568 (42.7)	94 (69.6)	837 (43.1)	112 (61.2)	1807 (43.4)	280 (65.0)		
Urinary tract infection	No	874 (97.8)	108 (96.0)	1293 (97.2)	130 (96.3)	1894 (97.5)	171 (93.4)	4061 (97.5)	409 (94.9)	0.002	0.484
	Yes	20 (2.2)	5 (4.4)	37 (2.8)	5 (3.7)	49 (2.5)	12 (6.6)	106 (2.5)	22 (5.1)		
Cytomegalovirus	No	870 (97.3)	106 (93.8)	1300 (97.7)	131 (97.0)	1858 (95.6)	171 (93.4)	4028 (96.7)	408 (94.7)	0.032	0.331
	Yes	24 (2.7)	7 (6.2)	30 (2.3)	4 (3.0)	85 (4.4)	12 (6.6)	139 (3.3)	23 (5.3)		
Leucopenia	No	889 (99.4)	111 (98.2)	1309 (98.4)	134 (99.3)	1937 (99.7)	182 (99.5)	4135 (99.2)	427 (99.1)	0.720	0.545
	Yes	5 (0.6)	2 (1.8)	21 (1.6)	1 (0.7)	6 (0.3)	1 (0.6)	32 (0.8)	4 (0.9)		
COVID-19	No					1940 (99.8)	182 (99.5)	4164 (99.9)	430 (99.8)	0.283	
	Yes					3 (0.2)	1 (0.6)	3 (0.1)	1 (0.2)		
LOHS, median (IQR)		35 (33)	48 (39)	35 (28)	64 (64)	33 (27)	58 (55)	34 (27)	56 (56)	0.001	0.004
IHM, n (%)		189 (21.2)	57 (50.4)	162 (12.2)	35 (25.9)	203 (10.4)	36 (19.7)	554 (13.3)	128 (29.7)	<0.001	<0.001

HAP: hospital-acquired pneumonia. SD: standard deviation. CCI: Charlson comorbidity index. CCI applies to different disease categories, the scores of which are added to obtain an overall score for each patient. We have grouped patients into three categories: low CCI (patients with no previously recorded disease), medium CCI (patients with one category), and high CCI (patients with two or more disease categories). LOHS: length of hospital stay. IQR: interquartile range. IHM: in-hospital mortality.

**Table 6:** Multivariate analysis of the factors associated with in-hospital mortality among patients who underwent a solid organ transplant in Spain from 2004 to 2021.

		Kidney	Liver	Heart	Lung
		OR (95% CI)	OR (95% CI)	OR (95% CI)	OR (95% CI)
Sex	Female	1.23 (1.04–1.46)	1.12 (0.98–1.27)	1.24 (1.04–1.48)	1.15 (0.96–1.37)
Age groups	<45 years	1	1	1	1
	45–54 years	2.11 (1.53–2.91)	0.91 (0.76–1.09)	0.99 (0.78–1.26)	1.22 (0.95–1.57)
	55–64 years	2.76 (2.04–3.72)	1.03 (0.87–1.22)	1.37 (1.12–1.69)	1.16 (0.93–1.46)
	≥65 years	4.73 (3.54–6.32)	1.63 (1.33–1.98)	1.40 (1.08–1.81)	0.96 (0.67–1.38)
Complications		2.27 (1.93–2.68)	3.22 (2.87–3.61)	1.10 (0.93–1.30)	1.12 (0.94–1.32)
Urinary tract infection		0.42 (0.31–0.57)	0.82 (0.62–1.08)	0.64 (0.44–0.93)	0.74 (0.44–1.27)
*Escherichia coli*		0.49 (0.05–4.62)	1.39 (0.35–5.62)	0.69 (0.07–6.55)	0.32 (0.07–1.53)
*Klebsiella pneumoniae*		2.71 (0.42–17.64)	0.31 (0.07–1.36)	2.25 (0.88–5.78)	1.00 (0.35–2.84)
Other Gram-negative bacteria		2.51 (0.82–7.63)	0.90 (0.34–2.37)	1.45 (0.62–3.38)	0.99 (0.43–2.32)
*Pseudomonas aeruginosa*		1.93 (0.84–4.40)	1.78 (0.94–3.35)	3.15 (1.33–7.49)	1.03 (0.57–1.85)
*Staphylococcus aureus*		3.94 (0.73–21.38)	1.50 (0.60–3.71)	1.11 (0.26–4.76)	0.77 (0.36–1.65)
*Streptococcus pneumoniae*		0.27 (0.04–2.05)	1.87 (0.79–4.42)	1.18 (0.30–4.71)	1.18 (0.29–4.78)
Anaerobic bacteria		0.99 (0.06–15.21)	0.64 (0.07–5.57)		
Year	2004–2009	1	1	1	1
	2010–2015	0.65 (0.52–0.80)	0.80 (0.70–0.92)	0.92 (0.76–1.11)	0.47 (0.38–0.58)
	2016–2021	0.65 (0.53–0.79)	0.73 (0.63–0.84)	0.72 (0.59–0.87)	0.39 (0.31–0.47)
Hospital-acquired pneumonia		4.47 (2.85–9.08)	2.96 (2.34–3.75)	1.86 (1.34–2.57)	2.97 (2.24–3.94)

OR: odds ratio. 95% CI: 95% confidence interval.

## Data Availability

According to the contract signed with the Spanish Ministry of Health and Social Services, which provided access to the databases from the Spanish National Hospital Database (Registro de Actividad de Atención Especializada. Conjunto Mínimo Básico de Datos, Registry of Specialized Health Care Activities, minimum basic dataset), we cannot share the databases with any other investigator and have to destroy the databases once the investigation has concluded. Consequently, we cannot upload the databases to any public repository. However, any investigator can apply for access to the databases by filling out the questionnaire available at https://www.sanidad.gob.es/estadEstudios/estadisticas/estadisticas/estMinisterio/SolicitudCMBD.htm (accessed on 20 May 2023). All other relevant data are included in the paper.

## Supplementary Materials

The following supporting information can be downloaded at: https://www.mdpi.com/article/10.3390/jcm12175532/s1, Table S1: Microorganisms (bacterial) isolated in people who underwent a solid organ transplant in Spain from 2004 to 2021 with a code for hospital-acquired pneumonia (HAP).

## Appendix A. ICD-9-CM and ICD-10-CM Codes for Diagnoses and Procedures

Codes for hospital-acquired pneumonia: ICD-9-CM: 481–486 and 997.31; ICD10: J13-J18 and J95.851.

Codes for kidney transplant procedures: ICD-9-CM: 55.6, 55.61, and 55.69; ICD10: 0TYxxxx.

Complications of kidney transplant: ICD-9-CM: 996.81; ICD10: T86.1, T86.10, T86.11, T86.12, T86.13, and T86.19.

Codes for liver transplant procedures: ICD-9-CM: 50.5, 50.51, and 50.59; ICD10: 0FY0.

Complications of liver transplant: ICD-9-CM: 996.82; ICD10: T86.4, T86.40, T86.41, T86.42, T86.43, and T86.49.

Codes for heart transplant procedures: ICD-9-CM: 37.51; ICD10: 02YA0Zx.

Complications of heart transplant: ICD-9-CM: 996.83; ICD10: T86.2, T86.20, T86.21, T86.22, T86.23, T86.29, T86.290, and T86.298.

Codes for lung transplant procedures: ICD-9-CM: 996.84; ICD10: 0BYC, 0BYD, 0BYF, 0BYG, 0BYH, 0BYJ, 0BYK, 0BYL, and 0BYM.

Complications of lung transplant: ICD-9-CM: 33.50, 33.51, and 33.52; IC10: T86.81, T86.810, T86.10, T86.811, T86.812, T86.813, and T86.819.

## Appendix B. ICD-9-CM and ICD-10-CM Codes for Covariates

Clostridioides infections: ICD-9-CM:008.45; ICD10: A04.71 and A04.72.

Cytomegalovirus infection: ICD-9-CM:078.5; ICD10: B25.x.

Pneumonia: ICD-9-CM: 481–486 and 997.31; ICD10: J13-J18 and J95.851.

Urinary tract infection: ICD-9-CM:599.0; ICD10: N39.0.

Leucopenia: ICD-9-CM: 288.50, 288.51, and 288.59; ICD10: D72.810, D72.818, and D72.819.

Cirrhosis: ICD-9-CM: 571.2, 571.5, and 571.6; ICD10: K70.3x, K71.7, P78.81, K74.3, K74.4, K74.5, and K74.6x.

Hypogammaglobulinemia: ICD-9-CM:279.00; ICD10: D80.1.

COVID-19: ICD10: U07.1.

*Escherichia coli*: ICD-9-CM: 482.82; ICD10: J15.5.

*Haemophilus influenzae*: ICD-9-CM:482.2; ICD10: J14.

*Klebsiella pneumoniae*: ICD-9-CM: 482.0; ICD10: J15.

Legionella: ICD-9-CM: 482.84; ICD10: A48.1.

Unspecified Streptococcus: ICD-9-CM: 482.3; ICD10: J15.4.

Other Gram-negative bacteria: ICD-9-CM: 482.83; ICD10: J15.6.

*Pseudomonas aeruginosa*: ICD-9-CM: 482.1; ICD10: J15.1.

*Staphylococcus aureus*: ICD-9-CM: 482.41 and 482.42; ICD10: J15.211 and J15.212.

*Streptococcus pneumoniae*: ICD-9-CM: 481; ICD10: J13.

Anerobic bacteria: ICD-9-CM: 482.81; ICD10: J15.8.
