# Supplementary material for: Association between Hospital-Acquired Pneumonia and In-Hospital Mortality in Solid Organ Transplant Admissions: An Observational Analysis in Spain, 2004–2021
Source: J Clin Med. 2023 Aug 25;12(17):5532. doi: 10.3390/jcm12175532 (PMC10488258; doi:10.3390/jcm12175532)
Supplement: Supplementary file 1 [file jcm-12-05532-s001.zip › jcm-2517146-supplementary.pdf]

Table S1. Microorganisms (bacterial) isolated in people who underwent a solid organ transplant in Spain from 2004 to 2021 with a code for hospital acquired pneumonia (HAP).

**Microorganisms, n (%)**	**KIDNEY**	**LIVER**	**HEART**	**LUNG**	**TOTAL**
Streptococcus pneumoniae	30(6.2)	30(4.4)	11(3.7)	9(2.1)	77(4.1)
Staphylococcus aureus	7(1.4)	28(4.1)	9(3.0)	38(8.8)	80(4.3)
Nonspecified Streptococcus	0(0)	0(0)	0(0)	4(0.9)	4(0.2)
Haemophilus influenzae	5(1.0)	3(0.4)	4(1.3)	4(0.9)	16(0.9)
Pseudomonas aeruginosa	37(7.6)	57(8.3)	24(8.0)	66(15.3)	181(9.7)
Klebsiella pneumoniae	7(1.4)	23(3.4)	20(6.7)	18(4.2)	68(3.6)
Escherichia coli	6(1.2)	11(1.6)	5(1.7)	14(3.3)	36(1.9)
Legionella pneumophila	0(0)	0(0)	2(0.7)	0(0)	2(0.1)
Other gramnegative bacteria	22(4.5)	28(4.1)	28(9.3)	31(7.2)	106(5.7)
Anerobic bacteria	4(0.8)	8(1.2)	6(2.0)	6(1.4)	22(1.2)
